# Acoustic Absorption in 3D-Printed PLA Biocomposite Models: Influence of Grid, Trihexagonal, and Gyroid Infills and Biomass Reinforcement

**DOI:** 10.1021/acsaenm.6c00342

**Published:** 2026-06-12

**Authors:** Faouzia Tayari, Regina Silva, Tiago Brilhante, Isabel Cardoso, Pedro Pinto, Vânia Freitas, Rui Ribeiro, Artur Ferreira, Nuno Gama

**Affiliations:** † CICECO - Aveiro Institute of Materials, 56062 University of Aveiro, Aveiro 3810-193, Portugal; ‡ Amplitude Acoustics − Acústica e Vibração, Lda., Maia 4470-177, Portugal; § Lightenjin II − Indústria de Iluminação, Lda., Águeda 3750-064, Portugal

**Keywords:** 3D printing, sustainable materials, sound absorption, biomass residues, Johnson−Champoux−Allard (JCA) model

## Abstract

The demand for sustainable and high-performance acoustic materials is rapidly increasing as industries seek eco-friendly solutions for noise control and circular material design. Conventional manufacturing methods often limit the ability to optimize internal structures for sound absorption, whereas 3D printing offers unprecedented freedom to design and fabricate complex geometries with precision. In this study, cylindrical absorbers were produced by additive manufacturing using polylactic acid (PLA) and PLA biocomposites reinforced with biomass residues. Different infill architectures with relative densities of 10, 20, and 30% were systematically investigated to assess the combined effect of geometry, porosity, and biofiller reinforcement on acoustic performance. Normal incidence and quasi-random incidence sound absorption coefficients were evaluated using the Microflown In-situ Impedance Gun 630–20,000 Hz, and the Johnson–Champoux–Allard (JCA) model was used to extract key microstructural parameters and validate predictive accuracy. The results show that gyroid infill structures significantly enhance sound absorption, with coefficients increasing up to approximately 0.9 at high frequencies for the 30% infill PLA samples. Biomass-reinforced composites further improve performance, with PLA–coffee systems reaching absorption values close to 1.0 in the high-frequency range (around 20,000 Hz), compared to lower values observed in cork-based and neat PLA structures. Overall, the study demonstrates that both infill architecture and biomass reinforcement play a critical role in tuning the acoustic response of additively manufactured PLA composites.

## Graphical abstract

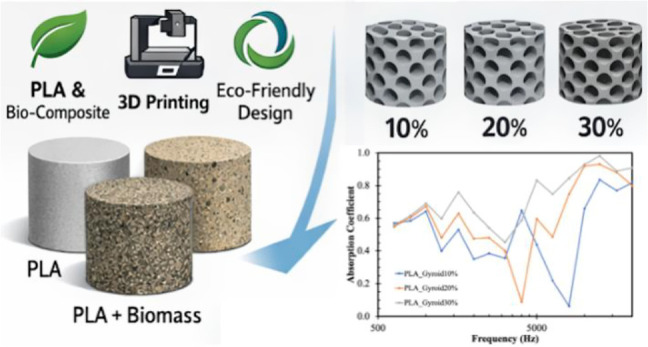

## 1 Introduction

Noise and vibration control is a critical challenge in fields such as architectural acoustics,^1–4^ transportation,^3^ and consumer product design.^3,5^ Prolonged exposure to excessive noise can lead to serious health issues,^5^ including hearing loss,^6^ cardiovascular problems,^6,7^ decreased productivity, and reduced overall well-being. Consequently, the development of effective sound-absorbing materials has become a key priority in modern engineering and design.^3,8,9^ Traditional absorbers, often based on petrochemical-derived foams or mineral fibers,^10–12^ provide effective performance but raise environmental concerns due to their low recyclability,^13,14^ limited biodegradability,^15^ and high energy consumption during production. In addition, these materials are often associated with toxic constituents or emissions, raising further health and environmental concerns. These drawbacks have prompted a growing interest in sustainable and high-performance alternatives.^13^

Biobased polymer composites manufactured through additive manufacturing (3D printing) are emerging as a promising solution for next-generation acoustic materials.^16–20^ Polylactic acid (PLA),^21^ a biodegradable polymer obtained from renewable resources such as corn starch or sugar cane,^22,23^ is particularly suitable for this purpose due to its environmentally friendly nature and excellent printability. Despite its mechanical strength, PLA faces limitations, hindering its widespread use in 3D printing techniques. To address this, recent studies focus on enhancing PLA through modifications or blending for 3D printing, strengthening its durability and heat resistance, and elevating its printability for a wide range of industrial uses.^24^ Arvinda Pandian et al.^25^ investigated FDM 3D printing of PLA/PBAT nanocomposites reinforced with graphene nanoplatelets, reporting enhanced printability and dimensional stability, although higher GnP contents led to reduced mechanical strength without significantly affecting hardness or thermal stability. Similarly, Mani et al.^26^ examined the effects of postprocessing treatments on 3D-printed PLA parts and demonstrated that isostatic compression at elevated temperatures significantly improved interlayer bonding and mechanical performance, resulting in notable increases in tensile, compressive, and flexural strengths for biodegradable and eco-friendly applications.

The capability of 3D printing to produce intricate internal structures, such as triply periodic minimal surface (TPMS) geometries, enables fine control over porosity and internal airflow pathways,^23,27^ which are critical parameters influencing acoustic absorption.^28,29^ Common TPMS geometries including gyroid, grid, and trihexagonal infills allows tailoring of the material’s performance across different frequency ranges. Incorporating biomass fillers such as cork and coffee residues into PLA matrices can further enhance acoustic performance.^30–32^ These natural fillers increase internal surface roughness,^30,31,33^ create additional microporosity,^34^ and promote more efficient energy dissipation when exposed to sound waves.^28,33,34^ Additionally, using biomass-derived fillers supports circular economy principles by valorizing industrial and agricultural waste streams.

To evaluate the efficiency of such materials, the sound absorption coefficient (α) is commonly used. This dimensionless parameter, ranging from 0 (perfect reflection) to 1 (perfect absorption),^35^ represents the fraction of incident acoustic energy absorbed by a surface. Experimental determination of α is typically carried out using impedance tube measurements, which provide reliable absorption data across a wide frequency range.^36^ Nevertheless, predicting the acoustic response of porous and biobased composites remains challenging due to the complex interplay of their heterogeneous structures with sound waves. To address this, several acoustic propagation models have been developed, ranging from empirical approaches to physically based approaches. Among these, the JCA model is one of the most widely used models.^37^ By incorporating parameters such as flow resistivity, porosity, tortuosity, and characteristic lengths, it provides a physically grounded framework for describing sound propagation in porous materials and enables accurate prediction of frequency-dependent absorption behavior.

Despite recent advances, a comprehensive understanding of how different infill geometries and biomass-derived fillers interact to influence the acoustic performance of 3D-printed PLA composites remains limited. Furthermore, the ability of existing acoustic propagation models to accurately predict the behavior of such complex materials is still not well-established. Therefore, this study aims to address these gaps by systematically analyzing the combined effects of infill geometry and biomass fillers on acoustic performance while also evaluating the applicability of current acoustic prediction models.

## 2 Materials and Methods

### 2.1 Materials

Virgin PLA (Fiberlogy), a neat filament without additives, was used as the reference material. In addition, two commercially available biocomposite filaments were used: PLA reinforced with coffee grounds (Francofil) and PLA reinforced with cork particles (Form Futura). Fourier transform infrared (FTIR) spectroscopy and X-ray diffraction (XRD) analyses of the filaments are presented in the Supporting Information. These materials were selected to evaluate the influence of biomass-derived fillers on the acoustic performance of 3D-printed structures while ensuring consistent processing conditions across all samples.

### 2.2 Sample Fabrication

All 3D-printed cylindrical samples, with a diameter of 200 mm and a height of 40 mm, were produced using a nozzle temperature of 200 °C and a bed temperature of 60 °C. Further information on the 3D printing parameters is provided in Table 1.

**Table 1:** 3D Printing Parameters

parameter	value
layer height	0.2 mm
wall thickness	0.8 mm
infill density	10/20/30%
printing temperature	205 °C
build plate temperature	45 °C
printing speed	50 mm·s^–1^

Three internal infill geometries were used: grid, trihexagonal, and gyroid. Furthermore, samples from each filament type were printed at three infill densities (10, 20, and 30%). To ensure reproducibility and enable accurate comparisons across different materials and geometries, all key printing parameters (including nozzle temperature, bed temperature, print speed, and layer thickness) were kept constant throughout the fabrication process. The final models are shown in Figure 1, highlighting the differences in geometry and infill density.

**Figure 1:**
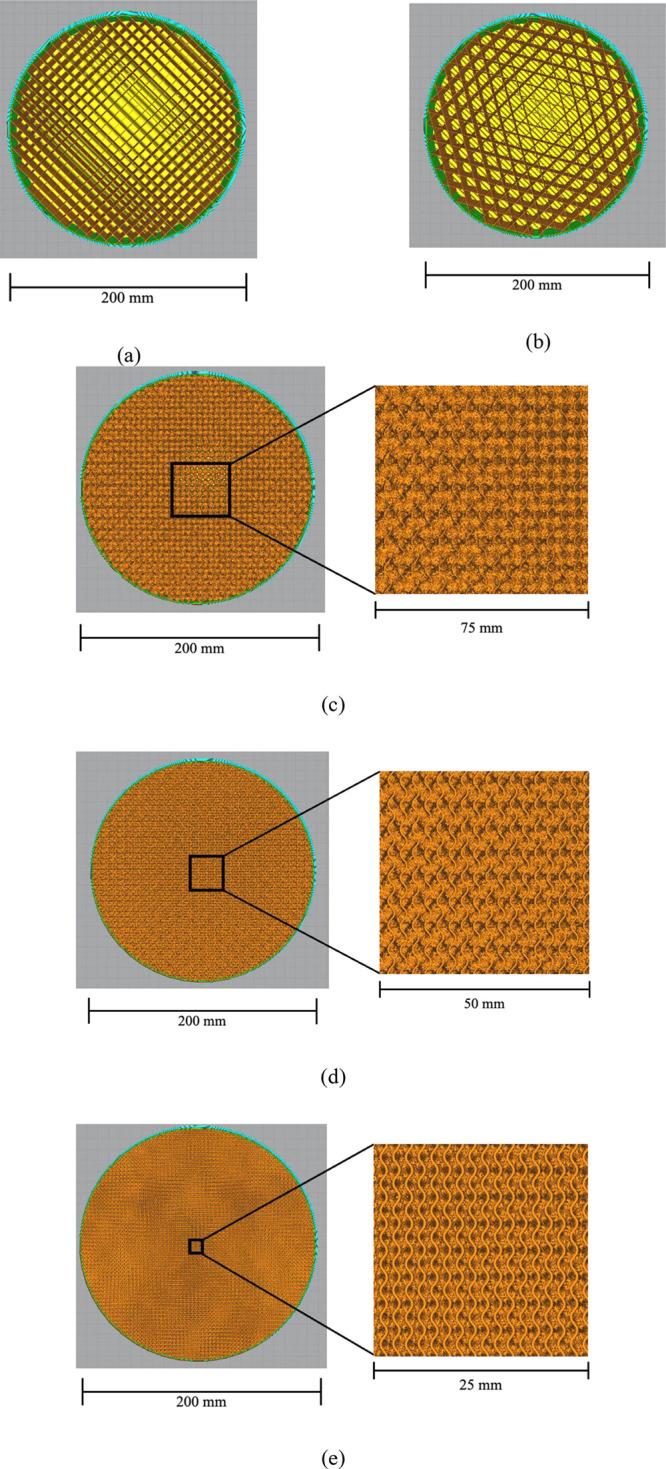
3D printing models: (a) grid10%; (b) trihexagonal10%; (c) gyroid10%; (d) gyroid20%; and (e) gyroid30%.

The resulting specimens, covering a range of material compositions, infill geometries, and densities, are summarized in Table 2.

**Table 2:** Details of the 3D-Printed Specimens

samples	materials	infill geometry	infill density (%)	porosity measured (ϕ)
(a) PLA_Grid10%	PLA	grid	10	0.89
(b) PLA_Trihexagonal10%	PLA	trihexagonal	10	0.89
(c) PLA_Gyroid10%	PLA	gyroid	10	0.89
(d) PLA_Gyroid20%	PLA	gyroid	20	0.79
(e) PLA_Gyroid30%	PLA	gyroid	30	0.68
(f) Cork_Gyroid10%	PLA + cork	gyroid	10	0.9
(g) Cork_Gyroid20%	PLA + cork	gyroid	20	0.81
(h) Coffee_Gyroid10%	PLA + coffee	gyroid	10	0.84

The porosity of the 3D-printed specimens was experimentally determined using a mass–volume method. First, each sample was accurately weighed using a precision balance to determine its mass. The corresponding external volume was calculated based on the specimen’s geometric dimensions. The density of the bulk PLA filament was used as a reference material property. Porosity was then calculated by comparing the apparent density of the printed structure (mass-to-volume ratio) with the density of the solid filament material. This approach allows the estimation of the overall void fraction of the printed structures. All 3D-printed models are shown in Figure 2.

**Figure 2:**
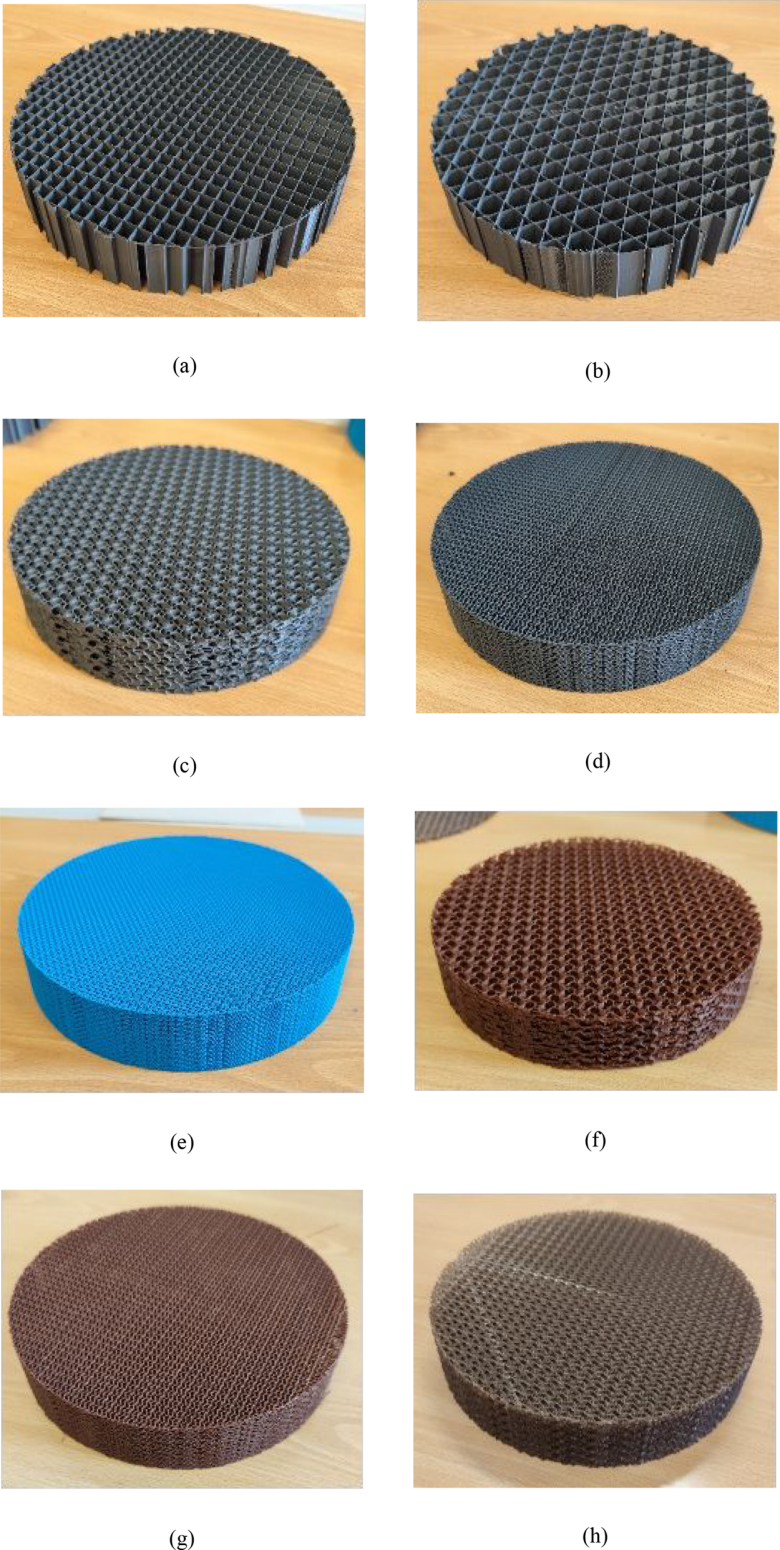
Images of the 3D-printed specimens: (a) PLA_Grid10%, (b) PLA_Trihexagonal10%, (c) PLA_Gyroid10%, (d) PLA_Gyroid20%, (e) PLA_Gyroid30%, (f) Cork_Gyroid10%, (g) Cork_Gyroid20%, and (h) Coffee_Gyroid10%.

### 2.3 Characterization

The FTIR spectra were collected on a PerkinElmer FTIR System Spectrum BX spectrometer equipped with a single horizontal Golden Gate ATR cell. All data were recorded at room temperature, in the range from 4000 to 600 cm^–1^ by accumulating 32 scans with a resolution of 4 cm^–1^. XRD patterns were collected using a Philips X’pert MPD diffractometer (Eindhoven, The Netherlands) using Cu Kα radiation. The scattered radiation was detected in the angular range from 5° to 50° (2θ).

The acoustic absorption properties of the 3D-printed samples were assessed using an in situ impedance measurement method with the Microflown Impedance Gun system,^38^ as presented in Figure 3.

**Figure 3:**
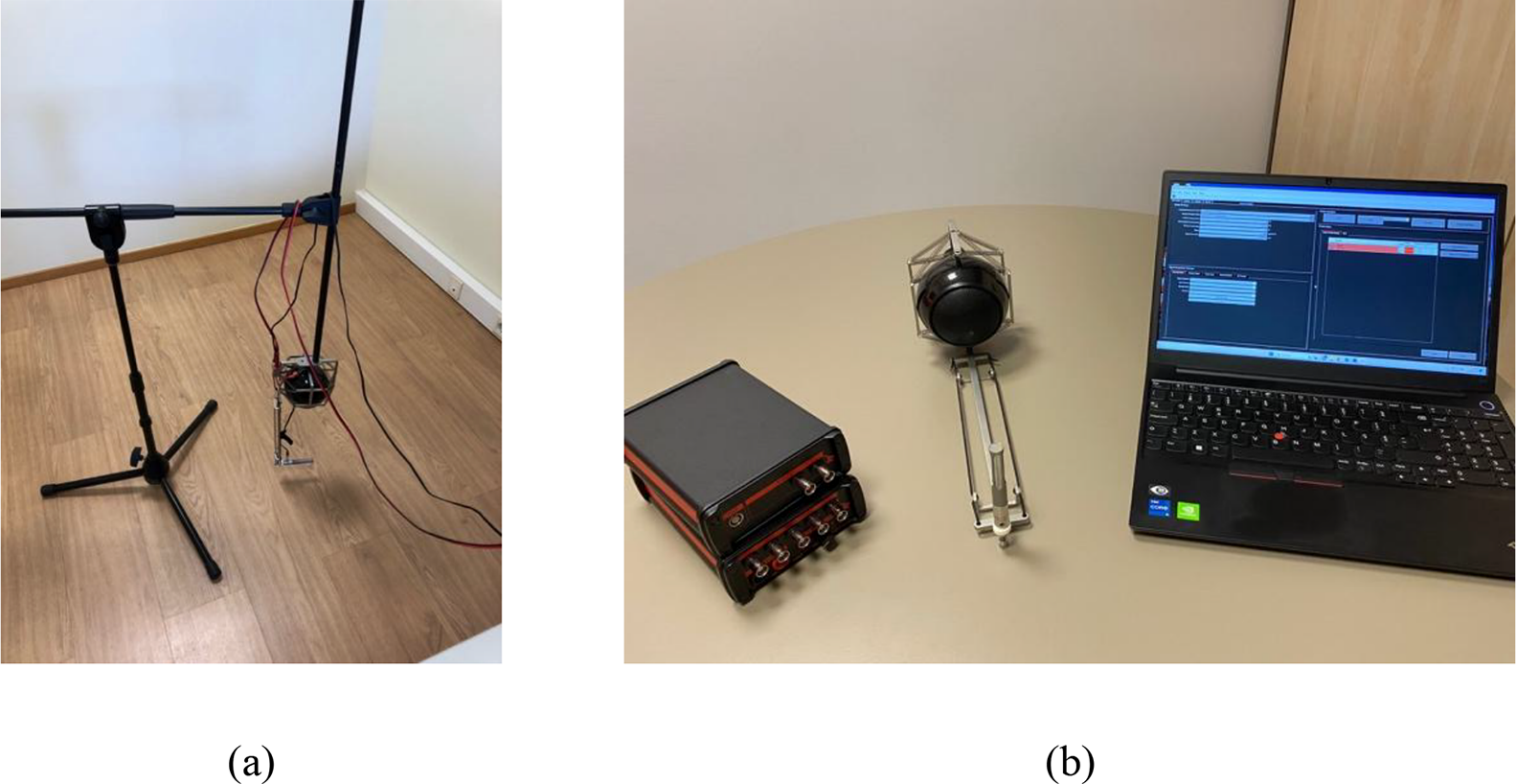
Setup of acoustic measurements using the in situ impedance measurement method consisting of a Microflown Impedance Gun (a) and a data acquisition system (b).

The tested specimens comprised both neat PLA and PLA-based composites reinforced with cork or coffee fillers, produced using different infill geometries (grid, trihexagonal, gyroid) and densities (10, 20, and 30%). The measurement system consists of a spherical loudspeaker mounted on a decoupled metallic frame and a PU probe (a PU probe is a dual-sensor acoustic measurement device that simultaneously measures sound pressure (*P*) using a microphone and particle velocity (*U*) using a particle velocity sensor, typically based on Microflown technology) positioned at a fixed distance from the source.^38,39^ The PU probe integrates an omnidirectional microphone placed near a particle velocity sensor, enabling the simultaneous acquisition of sound pressure and particle velocity. Tests were conducted in an empty office room of approximately 30 m^3^ characterized by bare plaster walls, a wooden floor, and acoustic ceiling tiles. The experimental setup included the Microflown In situ Impedance Gun, a tripod, a measuring tape, the Scout data acquisition system, the MFPA-2 signal conditioner, and a laptop running Velo software. The in situ impedance method offers several advantages, particularly for additively manufactured samples, as it enables nondestructive characterization of final parts without requiring additional sample preparation or a controlled laboratory condition.^40^ Measurements were performed under in situ quasi-random incidence conditions, which do not correspond to an ideal diffuse sound field.^41,42^ However, this technique is more sensitive to factors such as probe-to-sample distance, infill geometry, density, and sample size, which can significantly influence the results.^38–40^ Moreover, the compact design of the system imposes constraints on the reliable frequency range and on the derivation of surface impedance from the measured specific acoustic impedance.^43^ The absorption coefficient α­(*f*) was derived from the complex reflection coefficient *R*(*f*) measured by the surface impedance.^44^ The resulting absorption values are therefore interpreted as absorption indicators and used primarily for comparative analysis rather than as absolute random-incidence or diffuse-field coefficients.^41,42,45^ The absorption coefficient was calculated using eq 1:

$$\alpha (f)=1-|R(f){|}^{2}$$ 1

### 2.4 Johnson–Champoux–Allard (JCA) Model

The theoretical analysis of the acoustic behavior of the 3D-printed specimens was performed using the JCA model. Originally developed in the late 1990s, the JCA model has become a reference framework for characterizing sound propagation in rigid-frame porous materials, such as mineral wools, fibrous composites, and polymeric foams.^37,46,47^ Its strength lies in accounting for both viscous and thermal dissipation mechanisms within the pore structure, which makes it highly effective in predicting absorption over a wide frequency spectrum, particularly in the mid- to high-frequency range. In recent years, the model has been successfully extended to additively manufactured structures,^37,48^ where the pore network is artificially controlled by parameters such as infill geometry and density. This adaptability makes the JCA framework especially relevant for 3D-printed polymers and polymer–biomass composites, such as the PLA, PLA–cork, and PLA–coffee samples considered in the present study. The benefit of applying this model is that it not only provides a nondestructive and cost-effective tool for estimating acoustic behavior but also enables a direct link between design parameters (infill strategy, density) and functional performance. As such, the JCA approach forms a robust theoretical basis for evaluating the potential of sustainable 3D-printed composites in acoustic panel applications. In the JCA formulation, the effective density ρ_e_ and complex bulk modulus K_e_ are calculated using eqs 2 and 3, respectively:

$$\rho_{e}=\frac{\rho_{0}\alpha_{\infty }}{\phi }\left(1+\frac{\sigma \phi }{i\omega \rho_{0}\alpha_{\infty }}\sqrt{1+j\frac{4\alpha_{\infty }^{2}\eta \rho_{0}\omega }{\sigma^{2}\Lambda^{2}\phi^{2}}}\right)$$ 2

$$K_{e}=\frac{\gamma P_{0}}{\phi }{\left\{\gamma -(\gamma -1){\left[1+\frac{8\eta }{{{\mathrm{j\omega \Lambda}}^{\prime }}^{2}\rho_{0}N_{\mathrm{pr}}}{\left(1+j\frac{N_{\mathrm{pr}}\omega {\Lambda^{`}}^{2}\rho_{0}}{16\eta }\right)}^{1/2}\right]}^{-1}\right\}}^{-1}$$ 3

where ϕ is porosity, σ is flow resistivity (Pa·s/m^2^), α_∞_ is tortuosity (−), η is the dynamic viscosity of the air (Pa·s), Λ is the viscous characteristic length (m), Λ′ is the thermal characteristic length (m), *P*_0_ is atmospheric pressure (Pa), *P*_r_ is the Prandtl number, and ω is the angular frequency (rad/s).

## 3 Results and Discussion

The following section presents the characterization of the filaments used to fabricate the 3D-printed panels, in terms of their spectroscopic analysis, followed by an analysis of the measured sound absorption coefficients to examine how infill density, internal geometry, and biofiller additions affect the acoustic performance of panels. Data were grouped and compared based on controlled variations in sample design and composition.

### 3.1 Effect of Infill Density on Acoustic Absorption

The ensuing spectra are presented in Figure 4a,b.

**Figure 4:**
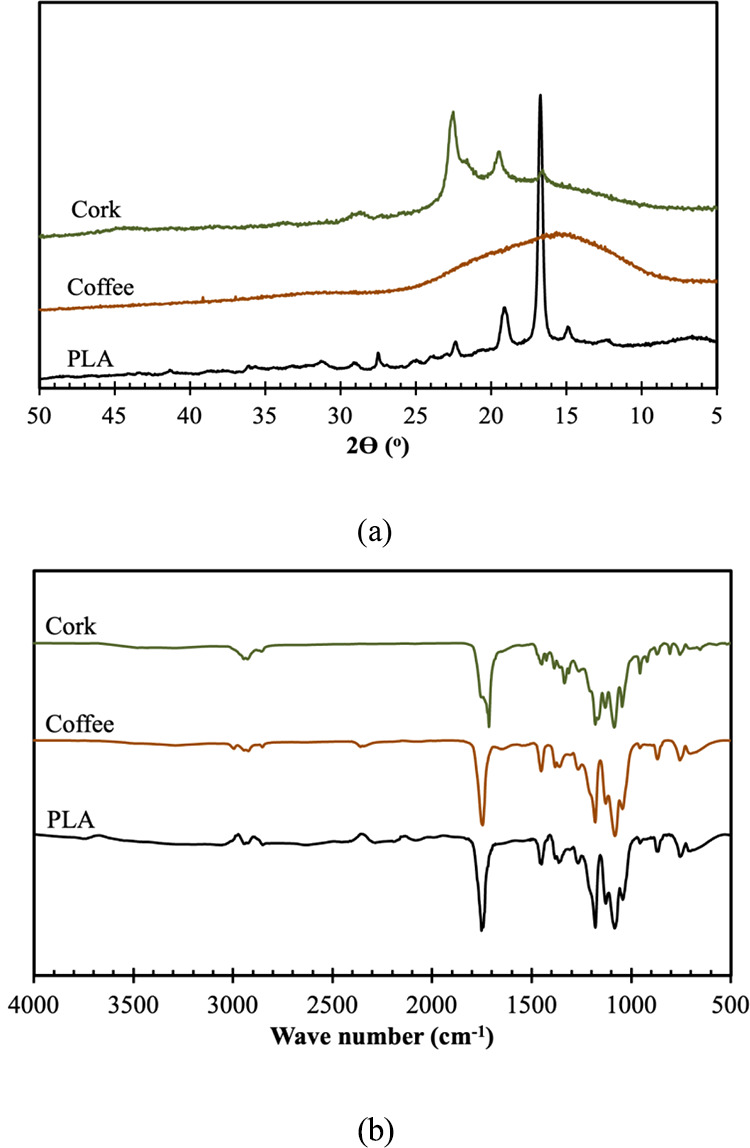
XRD (a) and FTIR (b) of filaments.

The XRD patterns of the three filaments used in this work, namely, PLA, PLA with cork, and PLA with coffee, presented in Figure 4a, reveal clear differences in their structural organization and degree of crystallinity. The neat PLA sample exhibits well-defined diffraction peaks, particularly a strong reflection around 2θ ≈ 16–17°, which is characteristic of the α-crystalline phase of PLA. Additional smaller peaks in the range of approximately 19–22° further confirm the presence of ordered crystalline domains. This indicates that the PLA filament is semicrystalline, with a relatively high degree of structural organization. When cork is incorporated into the PLA matrix, the diffraction pattern changes noticeably. The intensity of the characteristic PLA peaks decreases and the peaks become broader, especially in the region between 15° and 25°. This broadening is associated with an increase in the amorphous fraction and a reduction in crystal size or perfection. Cork, being mainly composed of amorphous components such as lignin and suberin, introduces structural disorder into the system. Although some minor features suggest that cork particles may act as heterogeneous nucleation sites, this effect is limited and does not compensate for the overall reduction in crystallinity. As a result, the PLA–cork composite exhibits a lower degree of crystallinity compared to neat PLA, with a more pronounced amorphous contribution. The PLA filament filled with coffee residues shows an even more significant change in its XRD profile. The pattern is dominated by a broad amorphous halo centered around 2θ ≈ 20°, and the distinct crystalline peaks of PLA are almost completely suppressed. This indicates that the presence of coffee residues strongly hinders the ability of PLA chains to organize into crystalline structures. Coffee waste is rich in amorphous organic compounds, which disrupt chain packing and may also act as physical barriers to crystallization. Compared to cork, coffee appears to have a weaker nucleating effect and a stronger tendency to promote amorphization of the matrix. Overall, the comparison shows a clear trend: neat PLA has the highest crystallinity, PLA with cork shows an intermediate structure with reduced but still detectable crystallinity, and PLA with coffee is predominantly amorphous. These structural differences are expected to influence the material properties significantly, with decreasing crystallinity generally leading to lower stiffness and thermal resistance but potentially higher ductility and improved processability.

### 3.2 Effect of Infill Density on Acoustic Absorption

To evaluate the effect of the infill density on acoustic absorption, the sound absorption response of PLA gyroid structures with varying infill densities is presented in Figure 5.

**Figure 5:**
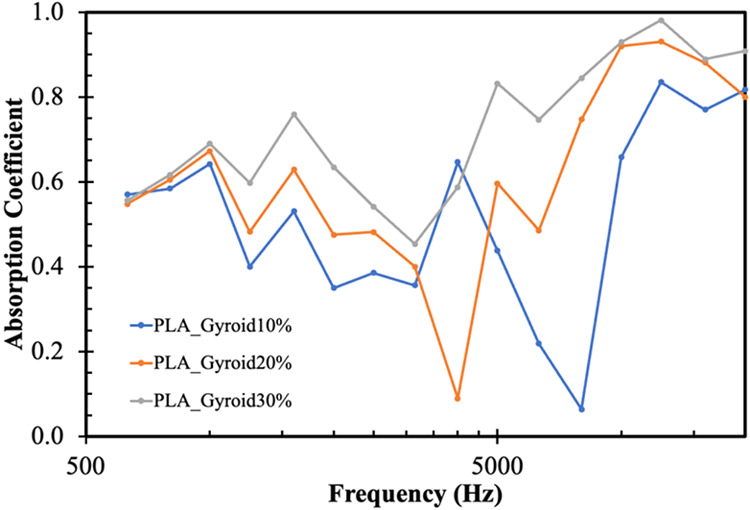
Sound absorption response of PLA gyroid structures with varying infill densities.

Overall, the absorption coefficient increases with frequency, and clear differences are observed between the three infill densities. At low-to-mid frequencies (630–3000 Hz), the 30% infill sample exhibits the highest absorption, reaching values of about 0.75 near 1000 Hz, while the 10 and 20% samples show lower values between 0.40 and 0.62. In the midfrequency region (3000–5000 Hz), the 30% infill sample presents a pronounced peak of approximately 0.83 at 5000 Hz, whereas the 10% sample maintains moderate absorption and the 20% sample shows a significant drop. At higher frequencies (above 5000 Hz), sound absorption becomes more effective for all samples, particularly for higher infill densities. The 30% gyroid structure demonstrates the highest and most stable performance with absorption coefficients close to 0.9, while the 20% sample reaches slightly lower peak values. In contrast, the 10% infill sample shows strong fluctuations, including a deep minimum followed by a sharp increase at higher frequencies. These results indicate that increasing gyroid infill density enhances sound absorption,^49^ especially at high frequencies, due to the increased internal surface area and dominant visco-thermal losses dominating,^50^ in agreement with previous studies on porous polymer structures.^51^

### 3.3 Effect of Geometry on Acoustic Absorption

To evaluate the effect of geometry on acoustic absorption, the sound absorption response of PLA trihexagonal, grid, and gyroid infill structures at 10% infill density is presented in Figure 6.

**Figure 6:**
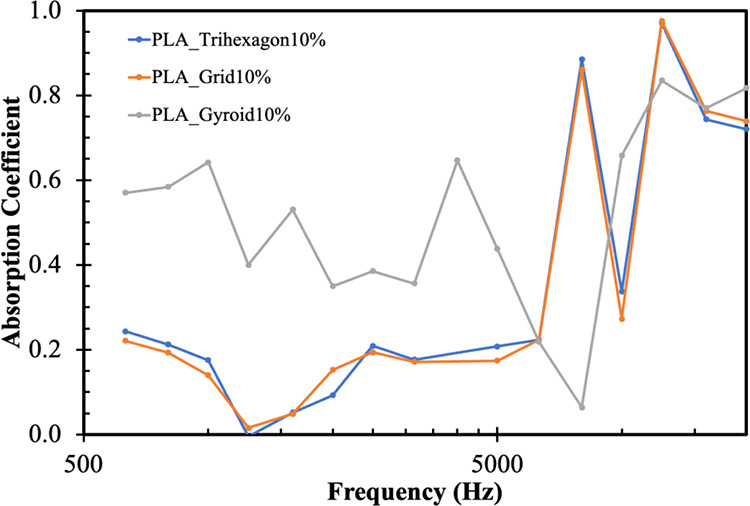
Sound absorption response of PLA trihexagonal, grid, and gyroid infill structures at 10% infill density.

Acoustic absorption was evaluated over the frequency range of approximately 630–20000 Hz, as shown in Figure 6, to investigate the influence of the internal geometry on sound absorption behavior. Across the lower-frequency region (below 1500 Hz), trihexagonal and grid structures exhibit relatively low absorption, with coefficients generally remaining below 0.25. In contrast, the gyroid geometry shows noticeably higher absorption, with values reaching approximately 0.55–0.65, indicating enhanced acoustic damping even at lower frequencies due to its complex internal architecture.^49^ At midfrequencies (approximately 1500–3000 Hz), the trihexagonal and grid structures display modest fluctuations in absorption, remaining mostly between 0.15 and 0.25, while the gyroid structure again exhibits higher absorption levels, reflecting increased visco-thermal and thermal losses associated with its interconnected pore network.^50^ At higher frequencies (above approximately 3000 Hz and extending up to 20,000 Hz), the trihexagonal and grid structures exhibit sharp absorption peaks, approaching 0.85–0.95, followed by a slight decrease at the upper end of the measured range. The gyroid structure also demonstrates strong high-frequency absorption, with coefficients exceeding 0.70–0.80, attributable to its high internal surface area,^52^ tortuosity,^53^ and multiple scattering paths that promote effective energy dissipation.^54^ Overall, the results indicate that internal geometry plays a critical role in frequency-dependent acoustic performance, with the gyroid geometry providing more consistent broadband absorption,^49^ while the simpler grid and trihexagonal geometries exhibit a more frequency-selective behavior.^55^

It should be noted that at the upper frequency limit (20,000 Hz), the acoustic wavelength in air (∼17 mm) becomes higher than the characteristic dimensions of the porous structure, particularly when considering the viscous characteristic length (∼2.07 mm for the 10% PLA gyroid). In this regime, the assumptions underlying the equivalent fluid (homogenized) approach begin to weaken, as local scattering effects and spatial nonuniformities may no longer be negligible. However, the JCA model remains generally valid as long as the wavelength is significantly larger than the characteristic pore scale, which is still largely satisfied in the present study. Therefore, the observed deviations at higher frequencies, especially for low-density or highly periodic structures, can be attributed to a gradual transition toward a weak scattering regime and a partial breakdown of the homogenization assumption.

### 3.4 Effect of Biomass Additions on Acoustic Absorption

To evaluate the effect of biomass additions on acoustic absorption, the sound absorption performance of 3D-printed PLA gyroid composites with biomass fillers (10% infill) as a function of frequency is presented in Figure 7.

**Figure 7:**
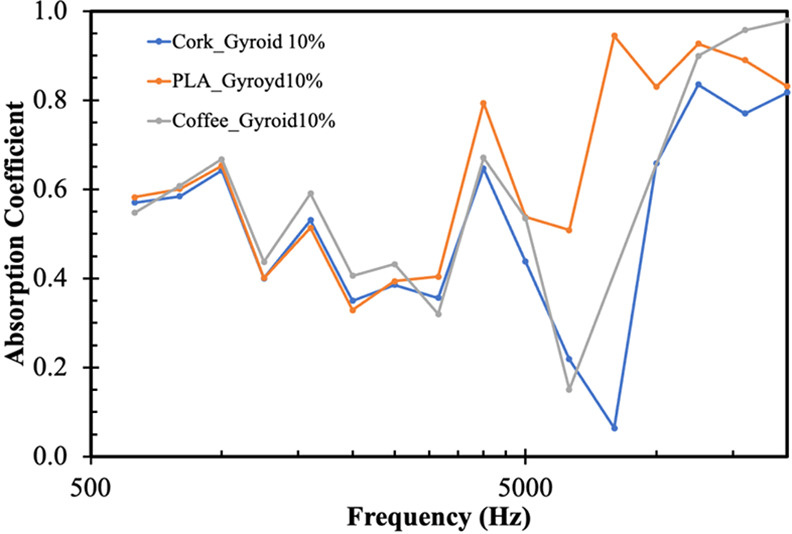
Sound absorption performance of 3D-printed PLA gyroid composites with biomass fillers (10% infill) as a function of frequency.

The sound absorption behavior of 3D-printed gyroid structures with 10% infill, produced using pure PLA and PLA reinforced with cork and coffee waste, is presented in Figure 7 over the frequency range from 630 to 20,000 Hz. Between 630 and 4000 Hz, all samples exhibit moderate absorption, with coefficients ranging from approximately 0.45 to 0.65, indicating comparable acoustic behavior. In the frequency range of 4000–5000 Hz, pure PLA shows the highest sound absorption among all samples, reaching values close to 0.8, while the PLA–coffee and the cork-filled samples exhibit slightly lower absorption, around 0.75 and 0.65, respectively. A pronounced absorption minimum occurs between 5000 and 6000 Hz for all materials, particularly for the cork composite, where the absorption coefficient drops below 0.1. At higher frequencies (above 6500 Hz), sound absorption increases sharply. The PLA–coffee composite achieves the highest absorption at the upper frequency limit, approaching values close to 1.0 near 20000 Hz. Pure PLA also exhibits strong high-frequency absorption, remaining slightly higher than the cork-filled sample, which reaches values around 0.85. These results indicate that pure PLA dominates the midfrequency acoustic response, while coffee grounds provide the most effective enhancement at high frequencies, highlighting the frequency-dependent acoustic performance of gyroid PLA structures.

## 4 Numerical Simulation and Acoustic Modeling

In this study, nonacoustic parameters such as tortuosity, flow resistivity, and characteristic lengths were not measured experimentally, being estimated using an inverse fitting approach based on the JCA model. Experimental sound absorption data were fitted using a nonlinear least-squares algorithm implemented in MATLAB, with initial parameter values taken from the literature.^56–59^ The frequency range of 1600–20,000 Hz was selected as it provides a reasonable compromise between fitting accuracy and physically realistic parameter estimation, where visco-thermal losses and microstructural effects are dominant and the JCA model remains valid. This approach enables reliable prediction of nonacoustic parameters from experimental data and supports a realistic interpretation of the influence of the type of structure, infill density, and biomass fillers on acoustic performance.

### 4.1 Fitted Absorption Curves Using the JCA

This section presents a comparison between experimentally measured and numerically predicted sound absorption coefficients obtained with the JCA model. The JCA model was selected because it remains one of the most widely used and physically grounded homogenized approaches for predicting sound absorption in porous materials, particularly in the mid- to high-frequency range. Although its fundamental assumptions (e.g., isotropy and absence of local resonances) are not fully satisfied in periodic lattice structures such as grid and trihexagonal geometries, it was still used to provide a consistent reference framework for all samples and to enable quantitative comparison across different types of structures, infill types, and biomass reinforcements.

Fitted curves were generated for pure PLA, PLA–coffee, and PLA–cork composites, produced using trihexagonal, grid, or gyroid structures, at infill densities of 10, 20, and 30%, across the frequency range of 1600–20,000 Hz, as presented in Figure 8. The results show that the JCA model can closely replicate the experimental absorption curves, thereby validating the fitted parameter set. Moreover, the simulations provide a deeper insight into how biomass fillers (cork and coffee) and internal geometry influence high-frequency acoustic absorption.

**Figure 8:**
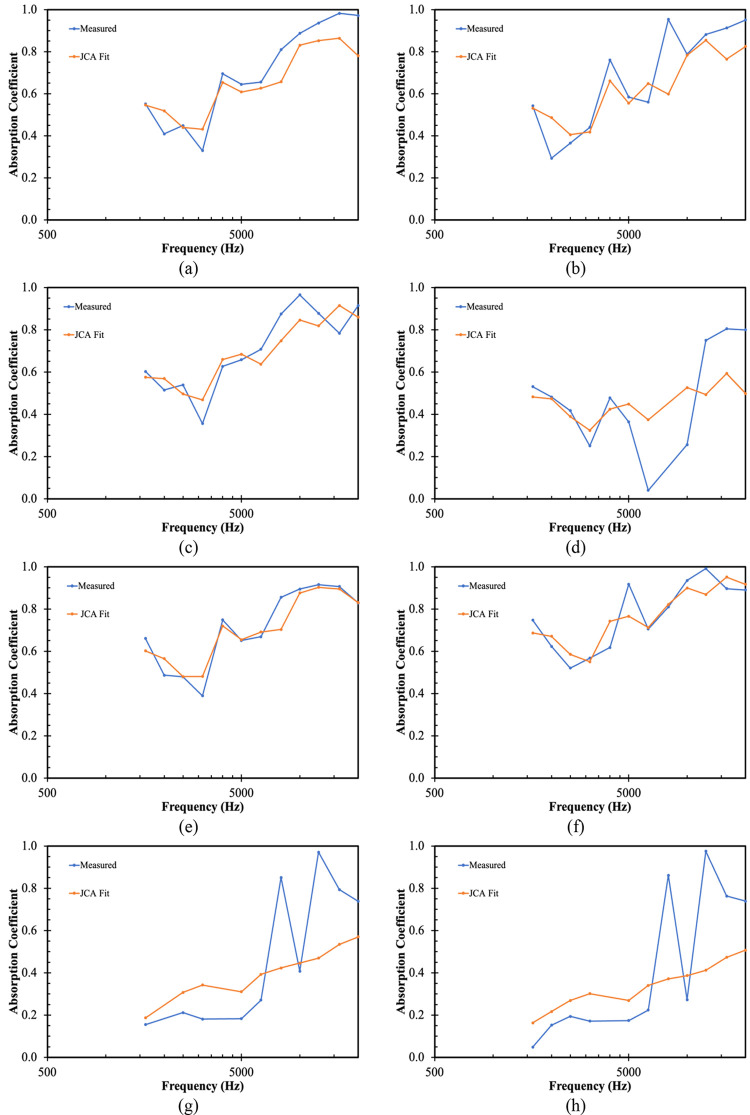
Comparison of measured and JCA-predicted acoustic absorption for 3D-printed PLA and biocomposite samples: (a) coffee_gyroid10% (*R* ^2^ = 0.805), (b) cork_gyroid10% (*R* ^2^ = 0.634), (c) cork_gyroid20% (*R* ^2^ = 0.792), (d) PLA_gyroid10% (*R* ^2^ = 0.321), (e) PLA_gyroid20% (*R* ^2^ = 0.882), (f) PLA_gyroid30% (*R* ^2^ = 0.775), (g) PLA_grid10% (*R* ^2^ = 0.340), and (h) PLA_trihexagonal10% (*R* ^2^ = 0.369).

As mentioned, Figure 8 compares the experimentally measured sound absorption coefficients with those predicted by the JCA model for 3D-printed PLA and PLA–biocomposite structures over the frequency range of 1600–20,000 Hz. Overall, the JCA model reproduces the general absorption trends reasonably well at mid- to high-frequency ranges,^47^ where visco-thermal dissipation dominates,^60^ although discrepancies remain in samples exhibiting strong local fluctuations or resonance effects. The coffee_gyroid10% sample (Figure 8a) shows high absorption above 5000 Hz, with good agreement between the measured and predicted curves (*R* ^2^ = 0.805), while the model slightly smooths the experimental peaks. Cork_gyroid10% (Figure 8b) exhibits a more gradual increase in absorption, but the agreement is weaker (*R* ^2^ = 0.634), reflecting the heterogeneous pore structure of cork.^61^ Increasing the cork content to 20% (Figure 8c) improves the overall fit (*R* ^2^ = 0.792), indicating enhanced viscous–thermal losses that are better captured by the JCA formulation.^45^ For pure PLA gyroid samples, the agreement strongly depends on infill density. PLA_gyroid10% (Figure 8d) displays pronounced fluctuations and a deep midfrequency dip, which the JCA model fails to reproduce accurately (*R* ^2^ = 0.321). In contrast, PLA_gyroid20% (Figure 8e) shows a smoother absorption response and excellent agreement with the model at mid-to-high frequencies (*R* ^2^ = 0.882). PLA_gyroid30% (Figure 8f) exhibits the most stable and highest absorption across the band, with good predictive performance of the JCA model (*R* ^2^ = 0.775). Periodic lattice geometries behave differently: PLA_Grid10% (Figure 8g) and PLA_Trihexagonal10% (Figure 8h) show sharp, geometry-driven absorption peaks that are significantly underestimated by the JCA model (*R* ^2^ is between 0.34 and 0.37), highlighting its limitations for resonance-dominated and anisotropic structures as similarly noted by Horoshenkov et al.^62^ and Dupont et al.^63^

Overall, these results confirm that the JCA model is well suited for predicting the mid-to-high-frequency absorption behavior of gyroid-based porous structures,^43,49^ particularly at higher infill densities and for biocomposites. Deviations between measurements and simulations are mainly attributed to structural heterogeneity, anisotropy, and resonance effects that are not fully accounted for in the homogenized modeling framework. In other words, the observed discrepancies for the grid and trihexagonal structures (*R* ^2^ = 0.34–0.37) are mainly attributed to geometry-induced anisotropy and resonance effects, which are not captured by the equivalent fluid approximation of the JCA model. In contrast, gyroid structures, which exhibit more isotropic and tortuous pore networks, are better aligned with the model assumptions and therefore show improved predictive accuracy.

### 4.2 Model Validation and Acoustic Parameter Correlation

To further validate the predictive capability of the JCA model, the coefficient of determination (*R* ^2^) was used as a quantitative indicator of the agreement between experimentally measured and numerically simulated absorption coefficients. The fitted acoustic parameters including tortuosity (α_∞_), flow resistivity, viscous characteristic length (Λ), and thermal characteristic length (Λ′) are summarized in Table 3 for the different material compositions and internal geometries.

**Table 3:** Fitted JCA Model Parameters and *R* ^2^ Values for 3D-Printed Structures^a^

samples	tortuosity α_∞_	flow resistivity σ (Pa·s/m^2^)	viscous length Λ (μm)	thermal length Λ′ (μm)	*R* ^2^
Coffee Gyroid10%	1.81	700	860.3	4730	0.80
Cork_Gyroid10%	1.92	700	865.0	2316	0.63
Cork_Gyroid20%	1.68	700	732.5	1278	0.79
PLA_Gyroid10%	1.77	1568.9	2070.3	5000	0.32
PLA_Gyroid20%	1.81	700	833.5	1378	0.88
PLA_Gyroid30%	1.65	1447.4	821.2	5000	0.75
PLA_Grid10%	1.00	700	884.2	869	0.34
PLA_Trihexagonal10%	1.00	700	729.6	991	0.36

a Infill types include grid, trihexagonal, and gyroid at 10, 20, and 30% density for different composite formulations.

Table 3 presents the acoustic parameters from fitting the JCA model to the 3D-printed samples' measured acoustic response. Gyroid samples show higher tortuosity (α_∞_ = 1.65–1.92) than grid and trihexagonal samples (α_∞_ = 1.0), reflecting more complex flow paths.^53,64^ Flow resistivity varies between 700 and 1569 Pa·s/m^2^, consistent with highly permeable polymer lattices.^58,65^ For very open structures, several samples converge near 700 Pa·s/m^2^, indicating limited sensitivity of the acoustic response to this parameter. The viscous characteristic length (Λ) ranges from 730 to 2070 μm, consistent with the millimeter-scale pore sizes determined by the printing process.^58,50^ Although viscous characteristic lengths are rarely reported explicitly in such materials, their magnitudes align with classical porous media theory relating viscous length to pore geometry.^45^ From Table 3, it can also be seen that Λ′ varies with both the material and the architecture. In the analyzed samples, structures with similar porosity show different Λ′ values, highlighting the key role of pore topology. Despite not following a linear correlation, the results suggest that more constrained geometries, such as gyroid, exhibit higher Λ′, while more open structures, such as trihexagonal designs, show lower values and stronger thermal interactions. Model fits vary (*R* ^2^ = 0.34–0.88), with the best agreement observed in gyroid structures of moderate density and biofiller content. Due to parameter coupling, the extracted values should be interpreted as effective macroscopic descriptors of the acoustic behavior rather than unique intrinsic properties.

Recent studies highlight the potential of additive manufacturing to develop lightweight, multifunctional structures for acoustic and structural applications. In particular, PLA-based biodegradable composites, such as PLA/PBAT blends, have been identified as promising alternatives to conventional petroleum-based polymers due to their improved toughness and reduced brittleness.^24^ Furthermore, the incorporation of secondary fillers, such as graphene nanoplatelets, further enhances material performance by improving energy dissipation, stress transfer, and damping capacity within the polymer matrix. Similar to this study, it was observed that the sound absorption behavior of 3D-printed composites was strongly governed by infill density and internal porosity, which directly influence wave propagation and visco-thermal losses. Lower infill densities tend to enhance sound absorption in the low- and midfrequency ranges due to increased porosity and wave penetration, whereas higher infill densities improve high-frequency absorption by promoting greater interaction between sound waves and the solid phase. Overall, the literature confirms that the acoustic performance of such systems is not controlled by a single parameter but rather by the coupled effect of material composition and internal architecture, highlighting the importance of simultaneous control of porosity, filler content, and structural design in the development of advanced acoustic materials.

## 5 Conclusions

This work demonstrates the effective characterization of the acoustic properties of 3D-printed PLA biocomposites with gyroid infill architectures reinforced with biomass residues, using the Microflown In situ Impedance Gun combined with the JCA model. Both gyroid geometry and biofiller reinforcement enhanced sound absorption by influencing key microstructural parameters, including porosity, tortuosity, and flow resistivity. The results indicate that gyroid infill structures substantially improve sound absorption, with coefficients reaching about 0.9 at high frequencies for PLA samples with 30% infill. The addition of biomass reinforcements further enhances this effect, with PLA–coffee composites achieving absorption values close to 1.0 in the high-frequency range (around 20000 Hz), outperforming both cork-based and neat PLA structures, which exhibit lower values. The inverse modeling approach further provided effective macroscopic acoustic descriptors that capture the complex absorption behavior of these materials with reasonable accuracy. In particular, gyroid structures with higher infill density exhibited the most stable and improved acoustic performance across the investigated frequency range. Overall, this study confirms the potential of combining additive manufacturing and biomass fillers to tailor the acoustic response of PLA-based materials for sustainable noise control applications.

## References

[1] Hemmat W., Hesam A. M., Atifnigar H. (2023). Exploring Noise Pollution, Causes,
Effects, and Mitigation Strategies: A Review Paper. European Journal of Theoretical and Applied Sciences.

[2] Sheikhmozafari M. J. (2021). Assessment of Noise Effect on Employee Comfort in an Open-Plan Office:
Validation of an Assessment Questionnaire. J.
Occup. Health Epidemiol..

[3] Arenas C., Leiva C., Vilches L. F., Cifuentes H. (2013). Use of Co-Combustion
Bottom Ash to Design an Acoustic Absorbing Material for Highway Noise
Barriers. Waste Management.

[4] Lim H., Utyuzhnikov S. V., Lam Y. W., Turan A. (2011). Multi-Domain Active
Sound Control and Noise Shielding. J. Acoust.
Soc. Am..

[5] Gupta D., Julka A., Jain S., Aggarwal T., Khanna A., Arunkumar N., de Albuquerque V. H.
C. (2018). Optimized Cuttlefish
Algorithm for Diagnosis of Parkinson’s Disease. Cogn. Syst. Res..

[6] Granberg S., Gustafsson J. (2021). Key Findings about Hearing Loss in
the Working-Life:
A Scoping Review from a Well-Being Perspective. Int. J. Audiol..

[7] Mehrotra A., Shukla S. P., Shukla A. K., Manar M. K., Singh S. K., Mehrotra M. (2024). A Comprehensive Review
of Auditory and Non-Auditory
Effects of Noise on Human Health. Noise Health.

[8] Kumar
Panda S., Charan Rath K., Mishra S., Khang A. (2023). Revolutionizing
Product Development: The Growing Importance of 3D Printing Technology. Mater. Today Proc..

[9] Opiela K. C., Zieliński T. G., Attenborough K. (2022). Limitations on Validating Slitted
Sound Absorber Designs through Budget Additive Manufacturing. Mater. Des..

[10] Whabi V., Yu B., Xu J. (2024). From Nature to Design:
Tailoring Pure Mycelial Materials
for the Needs of Tomorrow. Journal of Fungi.

[11] Setaki F., Tian F., Turrin M., Tenpierik M., Nijs L., van Timmeren A. (2023). 3D-Printed
Sound Absorbers: Compact
and Customisable at Broadband Frequencies. Architecture,
Structures and Construction.

[12] Liang J., Liu Z., Wang Q., Zhao Y., Fard M., Davy J. L. (2023). Optimization
of the Acoustic Performance of a Composite Multi-Cell Sound Absorber. Acoust. Aust..

[13] Buratti C., Belloni E., Lascaro E., Lopez G. A., Ricciardi P. (2016). Sustainable
Panels with Recycled Materials for Building Applications: Environmental
and Acoustic Characterization. Energy Procedia.

[14] Delany M. E., Bazley E. N. (1970). Acoustical Characteristics
of Fibrous Absorbent Material. J. Acoust. Soc.
Am..

[15] Mazur J., Sobczak P., Panasiewicz M., Łusiak P., Krajewska M., Findura P., Obidziński S., Żukiewicz-Sobczak W. (2025). Mechanical Properties and Biodegradability
of Samples Obtained by 3D Printing Using FDM Technology from PLA Filament
with By-Products. Sci. Rep..

[16] Calignano F. (2017). Overview on Additive
Manufacturing Technologies. Proc. IEEE.

[17] Calignano F., Manfredi D., Ambrosio E. P., Biamino S., Lombardi M., Atzeni E., Salmi A., Minetola P., Iuliano L., Fino P. (2017). Overview on Additive
Manufacturing Technologies. Proceedings of the
IEEE.

[18] Goh G. D., Yap Y. L., Agarwala S., Yeong W. Y. (2019). Recent
Progress
in Additive Manufacturing of Fiber Reinforced Polymer Composite. Adv. Mater. Technol..

[19] Suárez L. (2020). Assessment on the Use
of Additive Manufacturing Technologies for
Acoustic Applications. Int. J. Adv. Manuf. Technol..

[20] Suárez L., del Mar Espinosa M. (2020). Assessment
on the Use of Additive Manufacturing Technologies
for Acoustic Applications. International Journal
of Advanced Manufacturing Technology.

[21] Shah A. K., Jain A. (2024). Microstructure and
Mechanical Properties of Filament and Fused Deposition
Modelling Printed Polylactic-Acid and Carbon-Fiber Reinforced Polylactic-Acid. Journal of Reinforced Plastics and Composites.

[22] Mukherjee C., Varghese D., Krishna J. S., Boominathan T., Rakeshkumar R., Dineshkumar S., Brahmananda Rao C. V. S., Sivaramakrishna A. (2023). Recent Advances in Biodegradable
Polymers – Properties, Applications and Future Prospects. Eur. Polym. J..

[23] Siddiqui M. A. S., Rabbi M. S., Ahmed R. U., Billah Md. M. (2024). Biodegradable Natural
Polymers and Fibers for 3D Printing: A Holistic Perspective on Processing,
Characterization, and Advanced Applications. Cleaner Materials.

[24] Pandian C. K. A., Muthaiah T., Purushothaman R., Subramanian J. (2025). Evaluating
the Acoustic and Low-Velocity Impact Behavior of Sustainable Polylactic
Acid (PLA)/Poly­(Butylene Adipate-Co-Terephthalate) (PBAT)/Graphene
Nanoplatelet Three-Dimensional (GNP 3D) Printed Composites. Proceedings of the Institution of Mechanical Engineers, Part
E: Journal of Process Mechanical Engineering.

[25] Arvinda
Pandian C. K., Thirumurugan M., Shahitha Parveen J., Rajesh G. (2025). 3D Fused Deposition Modelling of PLA/PBAT Nanocomposites
Reinforced with GnP: Mechanical and Thermal Characterizations. J. Elastomers Plast..

[26] Mani S., Kasi A., Guruswamy R., Nilagiri Balasubramanian K. B., Pandian A. (2023). Effect of Post-Processing
Treatment on 3D-Printed Polylactic
Acid Parts: Layer Interfaces and Mechanical Properties. Int. J. Mater. Res..

[27] Rashid F. L., Al Maimuri N. M. L., Al-Obaidi M. A., Eleiwi M. A., Ameen A., Ahmad S., Chibani A., Kezzar M., Agyekum E. B. (2025). Enhancing
Heat Transfer across Applications with Triply Periodic Minimal Surface
(TPMS) Structures: A Comprehensive Review. Chemical
Engineering and Processing - Process Intensification.

[28] Berardi U., Iannace G. (2015). Acoustic Characterization
of Natural Fibers for Sound
Absorption Applications. Build. Environ..

[29] Xu Y., Li Y., Zhang A., Bao J. (2017). Epoxy Foams with Tunable Acoustic
Absorption Behavior. Mater. Lett..

[30] Duan Y., Chen X., Xia H., Liu Y., You F., Jiang X., Ren L., Zhou D. (2025). Fabrication,
Structural
Regulation and Future Applications of Acoustic Polymer Materials:
A Review. Appl. Mater. Today.

[31] Mohammadi M., Taban E., Tan W. H., Che Din N. B., Putra A., Berardi U. (2024). Recent Progress in
Natural Fiber Reinforced Composite
as Sound Absorber Material. Journal of Building
Engineering.

[32] Maskery I., Sturm L., Aremu A. O., Panesar A., Williams C. B., Tuck C. J., Wildman R. D., Ashcroft I. A., Hague R. J. M. (2018). Insights
into the Mechanical Properties of Several Triply Periodic Minimal
Surface Lattice Structures Made by Polymer Additive Manufacturing. Polymer (Guildf)..

[33] Taban E., Khavanin A., Faridan M., Samaei S. E., Samimi K., Rashidi R. (2020). Comparison of Acoustic Absorption
Characteristics of
Coir and Date Palm Fibers: Experimental and Analytical Study of Green
Composites. International Journal of Environmental
Science and Technology.

[34] Dou H., Cheng Y., Ye W., Zhang D., Li J., Miao Z., Rudykh S. (2020). Effect of
Process Parameters on Tensile
Mechanical Properties of 3D Printing Continuous Carbon Fiber-Reinforced
PLA Composites. Materials.

[35] Khosravani M. R., Reinicke T. (2021). Experimental Characterization
of 3D-Printed Sound Absorber. European Journal
of Mechanics - A/Solids.

[36] Corredor-Bedoya A. C., Acuña B., Serpa A. L., Masiero B. (2021). Effect of the Excitation
Signal Type on the Absorption Coefficient Measurement Using the Impedance
Tube. Applied Acoustics.

[37] Yi W., Guo J., Zhou T., Jiang H., Fang Y. (2024). A Machine Learning
Approach for Predicting the Johnson-Champoux-Allard Parameters of
a Fibrous Porous Material. Applied Acoustics.

[38] Briere
de La Hosseraye B., Yang J., Hornikx M. (2024). In Situ Acoustic Characterization
of a Perforated Panel on a Cavity by Means of PU Measurement and Model
Fitting. Building Acoustics.

[39] Briere
de La Hosseraye B., Hornikx M., Yang J. (2022). In Situ Acoustic Characterization
of a Locally Reacting Porous Material by Means of PU Measurement and
Model Fitting. Applied Acoustics.

[40] Sturm L. D., Albakri M. I., Tarazaga P. A., Williams C. B. (2019). In Situ Monitoring
of Material Jetting Additive Manufacturing Process via Impedance Based
Measurements. Addit. Manuf..

[41] Vorländer M., Mommertz E. (2000). Definition and Measurement
of Random-Incidence Scattering
Coefficients. Applied Acoustics.

[42] Dupont S., Sanalatii M., Melon M., Robin O., Berry A., Le Roux J.-C. (2023). Measurement
of the Diffuse Field Sound Absorption Using
a Sound Field Synthesis Method. Acta Acustica.

[43] Allard, J. F. ; Atalla, N. Propagation of Sound in Porous Media; Wiley, 2009.

[44] Hald J., Song W., Haddad K., Jeong C.-H., Richard A. (2019). In-Situ Impedance
and Absorption Coefficient Measurements Using a Double-Layer Microphone
Array. Applied Acoustics.

[45] Dupont S., Sanalatii M., Melon M., Robin O., Berry A., Le Roux J.-C. (2022). Characterization
of Acoustic Materials at Arbitrary
Incidence Angle Using Sound Field Synthesis. Acta Acustica.

[46] Champoux Y., Allard J.-F. (1991). Dynamic Tortuosity
and Bulk Modulus in Air-Saturated
Porous Media. J. Appl. Phys..

[47] Johnson D. L., Koplik J., Dashen R. (1987). Theory of
Dynamic Permeability and
Tortuosity in Fluid-Saturated Porous Media. J. Fluid Mech..

[48] Lomte A., Xue Y., Johnston W., Song G., Bolton J. S., Sharma B. (2024). Acoustic Modeling
of Three-Dimensional-Printed Fibrous Sound Absorbers. J. Acoust. Soc. Am..

[49] Godakawela J., Lomte A., Sharma B. (2025). Sound Absorption
in Uniform and Layered
Gyroid and Diamond Triply Periodic Minimal Surface Porous Absorbers. Applied Acoustics.

[50] Yang Y., Liu C., Shi F., Yang Y., Liu L. (2025). Influence of Thermal
and Viscous Effects on Acoustic Energy Transmission Characteristics
in Microcolumn Arrays. Appl. Therm. Eng..

[51] Feng Z., Liu Y. (2025). The Latest Research
Status of Porous Sound-Absorbing Materials. Journal of Polymer Engineering.

[52] Leofanti G., Padovan M., Tozzola G., Venturelli B. (1998). Surface Area
and Pore Texture of Catalysts. Catal. Today.

[53] Sadouki M. (2018). Experimental
Measurement of the Porosity and the Viscous Tortuosity of Rigid Porous
Material in Low Frequency. Journal of Low Frequency
Noise, Vibration and Active Control.

[54] Tourin A., Fink M., Derode A. (2000). Multiple Scattering
of Sound. Waves in Random Media.

[55] Marabello G., Chairi M., Di Bella G. (2024). Optimising
Additive Manufacturing
to Produce PLA Sandwich Structures by Varying Cell Type and Infill:
Effect on Flexural Properties. Journal of Composites
Science.

[56] Rong N., Min H., Tian H., Zhang R., Wang Z., Yue W. (2025). Simultaneous
Inverse Characterization of Five Non-Acoustic Parameters for Rigid
Porous Materials through Normal Surface Acoustic Impedance Measurements
in Impedance Tubes. Mech. Syst. Signal Process..

[57] Doutres O., Atalla N. (2011). Experimental Estimation
of the Transmission Loss Contributions
of a Sound Package Placed in a Double Wall Structure. Applied Acoustics.

[58] Panneton R., Olny X. (2006). Acoustical Determination
of the Parameters Governing Viscous Dissipation
in Porous Media. J. Acoust. Soc. Am..

[59] Allard, J. F. ; Atalla, N. Propagation of Sound in Porous Media: Modelling Sound Absorbing Materials. In Propagation of Sound in Porous Media: Modelling Sound Absorbing Materials; Springer, 2009; pp 1–358.

[60] Marabello G., Chairi M., Di Bella G. (2024). Optimising
Additive Manufacturing
to Produce PLA Sandwich Structures by Varying Cell Type and Infill:
Effect on Flexural Properties. J. Compos. Sci..

[61] Yay, Ö. ; Hasanzadeh, M. ; Diltemiz, S. F. ; Gürgen, S. Cork Agglomerates in Acoustic Insulation; Springer, 2024; pp 17–30.

[62] Horoshenkov K. V., Nichols A., Tait S. J., Maximov G. A. (2013). The Pattern of Surface
Waves in a Shallow Free Surface Flow. J. Geophys.
Res. Earth Surf..

[63] Dupont T., Leclaire P., Panneton R., Umnova O. (2018). A Microstructure Material
Design for Low Frequency Sound Absorption. Applied
Acoustics.

[64] Melkisedek B. L., Emeliana Y., Dharmawan I. A. (2025). A Comparative Study of Three-Dimensional
Flow Based, Geometric, and Empirical Tortuosity Models in Carbonate
and Sandstone Reservoirs. Applied Sciences.

[65] Embleton T. F. W., Piercy J. E., Daigle G. A. (1983). Effective
Flow Resistivity of Ground
Surfaces Determined by Acoustical Measurements. J. Acoust. Soc. Am..

